# Blockade of integrin α3 attenuates human pancreatic cancer via inhibition of EGFR signalling

**DOI:** 10.1038/s41598-019-39628-x

**Published:** 2019-02-26

**Authors:** Jungwhoi Lee, Jungsul Lee, Chulhee Choi, Jae Hoon Kim

**Affiliations:** 1 Department of Applied Life Science, SARI, Jeju National University, Jeju-do, 690-756 Republic of Korea; 2 Subtropical/tropical Organism Gene Bank, Jeju National University, Jeju-do, 690-756 Republic of Korea; 3 Department of Bio and Brain Engineering, KAIST, Daejeon, 34141 Republic of Korea; 4 Cellex Life Sciences Inc., Daejeon, 34141 Republic of Korea

## Abstract

The prognosis of pancreatic cancer remains dismal despite continuous and considerable efforts. Integrins (ITGs) are highly expressed in various malignant cancers. However, very few studies investigated the role of integrin α3 (ITGα3**)** in malignant cancers. Here, we determined the functional role of ITGα3 in pancreatic cancer. Analysis of public microarray databases and Western blot analysis indicated a unique expression of ITGα3 in human pancreatic cancer. Silencing ITGα3 expression significantly inhibited the viability and migration of human pancreatic cancer cells. Notably, ablation of ITGα3 expression resulted in a significant decrease of epidermal growth factor receptor (EGFR) expression compared with transfection of control-siRNA through an increased number of leucine-rich repeats and immunoglobulin-like domain protein 1 (LRIG1) expression. In addition, ablating ITGα3 inhibited tumour growth via blockade of EGFR signalling *in vivo*. Furthermore, the highly expressed ITGα3 led to a poor prognosis of pancreatic cancer patients. Our results provide novel insights into ITGα3-induced aggressive pancreatic cancer.

## Introduction

Pancreatic cancer is one of the most dangerous malignancies of the digestive system characterized by rapid progression, natural invasion, and grave patient outcome^1,2^. Despite continuous efforts to improve its prognosis, the incidence rates of pancreatic cancer are almost equal to its death rates^3^. Thus, there is an urgent need to develop effective therapeutics for this deadly neoplasm.

ITGs are primary transmembrane receptors that mediate cellular interactions with extracellular matrix (ECM) and regulate tumour cell features including adhesion, migration, invasion, proliferation, and survival^4–6^. The integrin receptor family consists of 18 α subunits and 8 β subunits that assemble as non-covalently connected heterodimers and organized into 24 different ITGs^7^. Our current *in silico* study and another previous report suggest that ITGα3 plays a significant role in adverse prognosis of pancreatic cancer^8,9^. However, the underlying mechanism is poorly understood.

Human epidermal growth factor receptor (EGFR) is a receptor tyrosine kinase (RTK) is characterized by an extracellular ligand-binding domain, a transmembrane portion, and a tyrosine kinase moiety^10^. Activation of EGFR signalling results in auto-phosphorylation of the tyrosine kinase domains, which amplify downstream signalling pathways such as mitogen-activated protein kinase (MAPK) and phosphatidylinositol 3-kinase (PI3K)/protein kinase B (AKT) pathway, leading to angiogenesis, growth, metastasis, and survival^11,12^. Due to mutations or over expression, inhibition of EGFR represents an assiduous therapeutic strategy via monoclonal antibodies (mAbs) and tyrosine kinase inhibitors (TKIs)^13^. In contrast, the predominant effects of negative signalling against EGFR in mammals prevailed for a long time, mediated by inducible feedback inhibitors (IFIs) such as leucine-rich repeats and immunoglobulin-like domain protein 1 (*LRIG1*), receptor-associated late transducer (*RALT*), suppressor of cytokine signalling 4 (*SOCS4*), and suppressor of cytokine signalling 5 (*SOCS5*) that act promptly without the need for de novo protein synthesis^14,15^. Various reports have highlighted the effects of IFIs on mammalian EGFR and demonstrated the critical role played by these molecules in the control of homeostasis^15^.

In the present study, we provide robust evidences suggesting that ITGα3 is critical for pancreatic malignancy via coordination with EGFR signalling pathway involved in alteration of LRIG1 expression. In addition, ITGα3 is specifically associated with adverse prognosis in pancreatic cancer. Our results establish a rationale for ITGα3 as a promising therapeutic target in patients with pancreatic cancer.

## Results

### Functional expression of integrin α3 (ITGα3) in human pancreatic cancers

**Figure 1:**
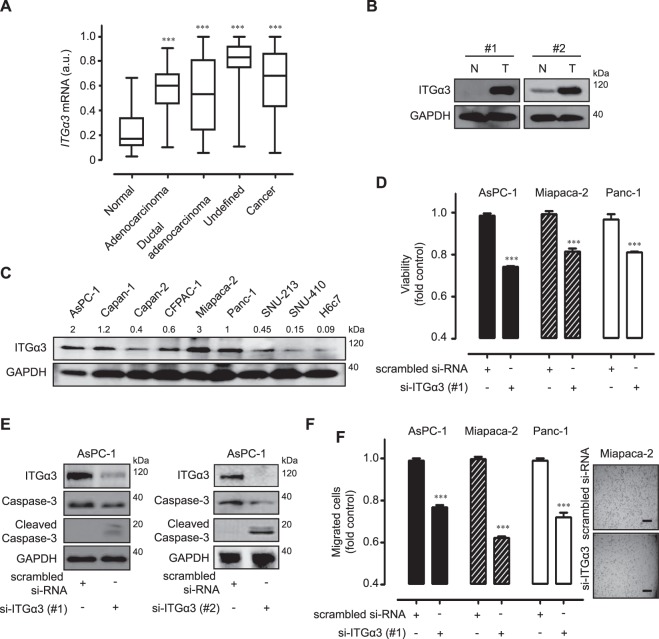
Functional integrin α3 (ITGα3) expression in pancreatic cancer. (**A**) Transcriptional levels of *ITGα3* in normal pancreas (*n* = 59, GSM# 388101–463724), adenocarcinoma (*n* = 55, GSM# 967641–1053825), ductal adenocarcinoma (*n* = 66, GSM# 388153–811004), and undefined samples (*n* = 76, GSM# 242823–414974) were analyzed using the Gene Expression Omnibus (GEO) databases (a.u. indicates arbitrary unit using the UPCs method, the *P* value was evaluated with Student’s *t-*test, ***P < 0.001, Cancer indicates the sum of adenocarcinoma, ductal adenocarcinoma, and undefined samples). (**B**) Protein expression of ITGα3 in pancreatic cancers and normal pancreas were analyzed using the Western blot. GAPDH was used as a control (N indicates a normal pancreas sample, T indicates a pancreatic cancer sample, #1 and #2 are separate samples, Data is representative of three individual experiments). (**C**) ITGα3 proteins in various human pancreatic cancer cells and H6c7 cells were detected by Western blot. GAPDH was measured as a control. Relative pixel intensities of ITGα3 were measured using ImageJ analysis software (ITGα3/GAPDH). Data is representative of three individual experiments. (**D**) AsPC-1, Miapaca-2, and Panc-1 cells were transfected with scrambled or ITGα3-specific siRNA for 72 h under serum-free cultured conditions. The viability was measured by WST-1 assay (*n* = 3; Tukey’s *post-hoc* test was used to detect significant differences in ANOVA, p < 0.0001; asterisks indicate a significant difference compared with 0% inhibition, ***P < 0.001). (**E**) AsPC-1 cells were transfected with scrambled or ITGα3-specific siRNAs (#1 and #2) for 72 h. ITGα3, caspase-3, and cleaved caspase-3 protein levels were analyzed by Western blot. GAPDH was used as a loading control. Data is representative of three individual experiments. (**F**) AsPC-1, Miapaca-2, and Panc-1 cells were transfected with scrambled or ITGα3-specific siRNA. After 48 h of transfection, the cells were exposed to serum-starved conditions. After 24 h of serum starvation, migrated cells were evaluated using the Transwell-migration assay (*n* = 3; Tukey’s *post-hoc* test was used to detect significant differences in ANOVA, p < 0.0001; asterisks indicate significant differences compared with 0% inhibition, ***P < 0.001, scale bar = 50 µm).

The expression profiles of *ITGα3* in human pancreatic cancer samples were obtained from public microarray database Gene Expression Omnibus (GEO). Adenocarcinoma of the pancreas, ductal-adenocarcinoma samples, and undefined cancers expressed higher levels of *ITGα3* than normal pancreas samples (Fig. 1A). To verify the expression patterns, we examined the ITGα3 protein levels in separate human pancreatic cancer tissues by Western blot analysis. ITGα3 was highly expressed in pancreatic cancer tissues compared with normal pancreas (Fig. 1B). In addition, ITGα3 was expressed relatively highly in eight human pancreatic cancer cells compared with human pancreatic duct epithelial H6c7 cells (Fig. 1C). To demonstrate the cellular functions of ITGα3, we inhibited ITGα3 expression by si-RNA transfection in ITGα3-expressing AsPC-1, Miapaca-2, and Panc-1 cells. Compared with AsPC-1, Miapaca-2, and Panc-1 cells transfected with control si-RNA, cells transfected with ITGα3-specific si-RNA showed significantly decreased levels of ITGα3 protein (Supplementary Fig. 1). Silencing of ITGα3 expression significantly inhibited the viability of AsPC-1, Miapaca-2, and Panc-1 cells under serum-free culture conditions (Fig. 1D). Similar result was obtained using another type of si-ITGα3 (#2) transfection (Supplementary Fig. 2A). Transfection using two different types of si-ITGα3 (#1 and #2) induced the caspase-3-mediated apoptosis (Fig. 1E). Ablation of ITGα3 expression also markedly diminished the migration of AsPC-1, Miapaca-2, and Panc-1 cells (Fig. 1F). At that time, there was no inhibition of viability between scrambled si-RNA or si-ITGα3 transfected cells (data not shown). Similar migration result was obtained using another type of si-ITGα3 (#2) transfection (Supplementary Fig. 2B). Correlations between *ITGα3* expression and various anti-cancer drugs were also demonstrated using Cancer Cell Line Encyclopedia (CCLE) public database to investigate the function of ITGα3 in human pancreatic cancer drug-resistance. *ITGα3* expression was negatively correlated with anti-cancer drug sensitivity in about 75% (18/24) of human pancreatic cancer cells (Table 1).

**Table 1:** Drug-sensitivities correlated with integrin α3 (ITGα3) expression levels in the CCLE databases.

Drug name	correlation	average	variation	N
Paclitaxel	−0.136521	0.7962	0.1585	28
RAF-265	−0.194086	0.8281	0.1089	23
17-AAG	0.007134	0.7962	0.1585	28
Irinotecan	−0.131134	0.8285	0.1199	19
Lapatinib	0.111679	0.7962	0.1585	28
Panobinostat	−0.165702	0.7962	0.1585	28
TKI258	−0.400488	0.7962	0.1585	28
Topotecan	0.166555	0.7962	0.1585	28
L-685458	−0.193066	0.7962	0.1585	28
PD-0332991	−0.161663	0.8295	0.1112	22
PF2341066	−0.020896	0.7962	0.1585	22
Erlotinib	−0.052117	0.7962	0.1585	28
AZD0530	−0.273753	0.7962	0.1585	28
LBW242	−0.049073	0.7962	0.1585	28
TAE684	−0.02831	0.7962	0.1585	28
ZD-6474	0.353902	0.7899	0.1580	28
PHA-665752	−0.379134	0.7962	0.1685	28
AZD6244	0.20628	0.7962	0.1585	28
PD-0325901	−0.070893	0.7962	0.1585	22
Sorafenib	−0.220974	0.7962	0.1585	28
Nilotinib	−0.07676	0.8290	0.1140	21
Nutlin-3	0.109441	0.7962	0.1585	28
PLX4720	−0.253	0.7899	0.1585	27
AEW541	−0.259067	0.7962	0.1585	28

Correlation of sensitivities to various anti-cancer drugs with ITGα3 expression levels in different pancreatic cancer cells.

^Ɨ^N indicates the number of human pancreatic cancer cell lines.

These results indicate that the functional expression of ITGα3 was elevated in human pancreatic cancer.

### Mechanism associated with integrin α3 (ITGα3) blockade in human pancreatic cancer cells

**Figure 2:**
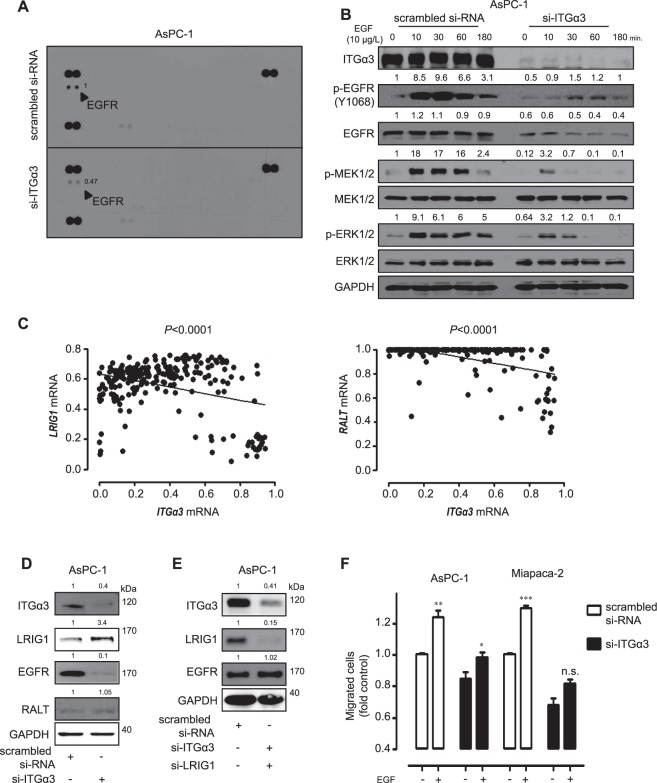
Associated mechanism following integrin α3 (ITGα3) blockade in human pancreatic cancer cells (**A**) AsPC-1 cells were transfected with scrambled or ITGα3-specific siRNA for 48 h. Human phospho-RTK array was used to determine differences in scrambled or ITGα3-specific siRNA transfection. Relative pixel intensity for p-EGFR was measured by densitometry analysis using ImageJ analysis software. Data is representative of two individual experiments. Bold arrows indicate the spot of EGFR. (**B**) AsPC-1 cells were transfected with scrambled or ITGα3-specific siRNA. After 48 h of transfection, the cells were exposed to serum-starved condition. After 18 h of serum starvation, AsPC-1 cells were incubated with 10 μg/L of EGF for various durations, and the cell lysates were subjected to Western blot using specific antibodies for p-EGFR (Y1068), EGFR, p-MEK1/2, MEK1/2, p-ERK1/2, ERK1/2, and GAPDH. Relative pixel intensities were measured by densitometry analysis using ImageJ analysis software. Data is representative of three individual experiments. (**C**) The correlation between *ITGα3* and *LRIG1* or *RALT* expression in the pancreatic cancer samples was calculated using the Gene Expression Omnibus (GEO) public microarray database (Pearson’s correlation coefficient (PCC) was used for statistical analysis). (**D**) AsPC-1 cells were transfected with scrambled or ITGα3-specific siRNA. After 72 h of transfection, the cell lysates were subjected to Western blot using antibodies specific for ITGα3, LRIG1, EGFR, RALT, and GAPDH. Relative pixel intensities were measured by densitometry using ImageJ analysis software. Data is representative of three individual experiments. (**E**) AsPC-1 cells were transfected with scrambled or combined ITGα3- and LRIG1-siRNA. After 72 h of transfection, the cell lysates were subjected to Western blot using antibodies specific for ITGα3, LRIG1, EGFR, and GAPDH. Relative pixel intensities were measured by densitometry using ImageJ analysis software. Data is representative of three individual experiments. (**F**) AsPC-1 and Miapaca-2 cells were transfected with scrambled or ITGα3-specific siRNA for 48 h. 10 μg/L of EGF was exogenously pretreated for 1 h. Migrated cells were evaluated using the Transwell-assay for a 6 h (*n* = 3; Tukey’s *post-hoc* test was used to detect significant difference in ANOVA, p < 0.0001; asterisks indicate a significant difference compared with 0% inhibition, *P < 0.05, **P < 0.01, ***P < 0.001, n.s. means non-significant).

Next, we examined signal transduction pathways using phospho-RTK array to delineate the mechanism by which ablation of ITGα3 expression affected features of human pancreatic cancer cells. Transfection with si-ITGα3 significantly reduced EGFR phosphorylation in AsPC-1 cells compared with control cells (Fig. 2A). To further analyze the detailed mechanism of ITGα3 knockdown, the levels of phosphorylated EGFR were examined with varying levels of exogenous EGF (10 µg/L) treatment following si-ITGα3 transfection in AsPC-1 cells. Treatment with exogenous EGF increased phosphorylation levels of EGFR (Tyr1068), MEK1/2 (Ser217/221), and ERK1/2 (Thr202/204) in AsPC-1 cells, but not in si-ITGα3 transfected cells (Fig. 2B). Notably, silencing of ITGα3 expression decreased the EGFR expression. To verify the reduction of EGFR expression by si-ITGα3 transfection, we examined the mRNA levels of *EGFR* following silencing of ITGα3 expression in AsPC-1 cells. Our results revealed that suppression of ITGα3 expression had no effect on *EGFR* mRNA expression level (Supplementary Fig. 3). Previous studies reported that inducible feedback inhibitors (IFIs) were natural inhibitors of EGFR expression^15,16^. To demonstrate the involvement of IFIs expression in *EGFR* down-regulation by reduction of ITGα3 expression, we initially examined the correlations between *IFIs* and *ITGα3* using the GEO public microarray database. A negative correlation was specifically found between *LRIG1* or *RALT* and *ITGα3* expression in pancreatic cancer samples (Fig. 2C). *SOCS4* and *SOCS5* showed a statistically non-significant or positive correlation with *ITGα3* (Supplementary Fig. 4). To examine the alteration of LRIG1 or RALT expression based on ITGα3 level, we performed si-ITGα3 transfection in AsPC-1 cells. A decreased ITGα3 expression increased the level of LRIG1 expression in AsPC-1 cells, but not RALT (Fig. 2D).

To support this correlation of ITGα3, LRIG1, and EGFR expression, we performed the combined transfection with si-ITGα3 and si-LRIG1 in AsPC-1 cells. EGFR expression level was not altered following dual transfection of si-ITGα3 and si-LRIG1 (Fig. 2E). We also demonstrated the EGFR expression levels of eight human pancreatic cancer cells and H6c7 cells. Miapaca-2 had a most high EGFR expression level; in contrast, SNU-410 and H6C7 cells had relatively low EGFR expression patterns (Supplementary Fig. 5). Ablation of ITGα3 expression also significantly diminished the migration potential of AsPC-1 and Miapaca-2 cells stimulated by exogenous EGF treatment (Fig. 2F). These results clearly indicate that ITGα3 ablation decreases EGFR signalling via induction of LRIG1 expression *in vitro*.

### *In vivo* effects of ITGα3 expression on human pancreatic cancer

**Figure 3:**
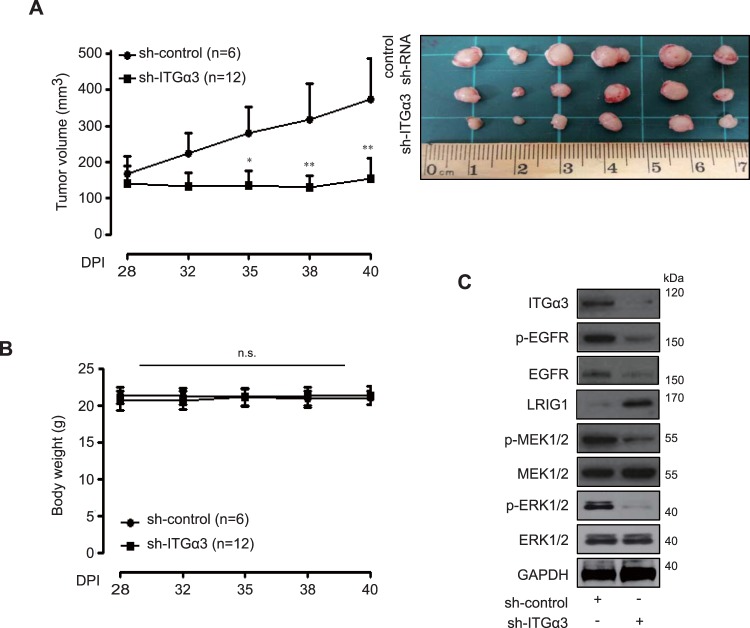
*In vivo* effects of integrin α3 (ITGα3) expression. (**A**) Anti-tumour effects of sh-ITGα3 AsPC-1 xenograft models (sh-control group: *n* = 6, sh-ITGα3 group: *n* = 12) were measured for 40 days using the formula: V = 0.523 LW^2^ (L = length, W = width) (Tukey’s *post-hoc* test was used to detect significant differences in ANOVA, *p* < 0.0001; asterisks indicate a significant difference compared with 0% inhibition, *p < 0.05, **p < 0.01 compared with sh-control group and sh- ITGα3 group). (**B**) Body weight in each group was regularly measured. (**C**) Western blot analysis of sh-control group and sh-ITGα3 group tumour lysate was conducted with anti ITGα3, p-EGFR, EGFR, LRIG1, p-MEK1/2, MEK1/2, p-ERK1/2, and ERK1/2 antibodies. GAPDH was used for loading control. Data is representative of three individual experiments.

To validate the effect of ITGα3 expression *in vivo*, we constructed a stably reduced sh-ITGα3 AsPC-1 cell line. We first determined the expression levels and features of the established cell line *in vitro*. Sh-ITGα3 AsPC-1 cells showed a significantly decreased expression of ITGα3 accompanied by reduced proliferation and migration activities under serum-free conditions (Supplementary Fig. 4). The effect of decreased expression of ITGα3 was then observed in sh-ITGα3 and sh-control AsPC-1 xenograft models. Control tumours grew to a mean size of 374.19 ± 113.7 mm^3^ at 40 days after transplantation with sh-control AsPC-1. However, shRNA-reducible tumours showed a mean size of 153.86 ± 57.6 mm^3^ at 40 days after transplantation with sh-ITGα3 AsPC-1 (Fig. 3A). No weight loss was detected in the control or sh-ITGα3 group of AsPC-1 xenograft models (Fig. 3B). To investigate the *in vivo* roles of ITGα3, we performed Western blot analysis using sh-ITGα1 and sh-control xenograft tumour lysates. The levels of ITGα3, phospho-EGFR, EGFR, phospho-MEK1/2, and phospho-ERK1/2 were significantly decreased in sh-ITGα3 AsPC-1 xenografts compared with those in sh-control AsPC-1 xenografts. In contrast, LRIG1 was markedly increased in sh-ITGα3 AsPC-1 xenografts compared with those in sh-control AsPC-1 xenografts (Fig. 3C).

**Figure 4:**
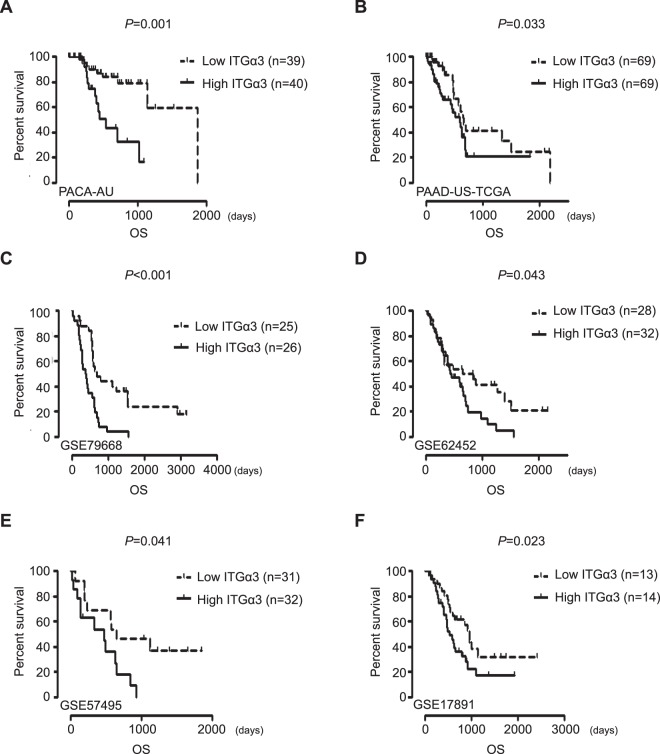
Integrin α3 (ITGα3) expression is associated with prognosis of pancreatic cancer patients. (**A**–**F**) Overall survival of pancreatic cancer patients was analyzed using Kaplan-Meier curves depending on the differential expression of ITGα3 in PACA-AU, PAAD-US-TCGA, GSE79688, GSE62452, GSE57495, and GSE17891 datasets (*P* value was calculated using Log-rank (Mantel-Cox) Test.

To demonstrate the role of ITGα3 in the prognosis of pancreatic cancer patients, we analyzed GEO datasets. PACA-AU, PAAD-US-TCGA, GSE79688, GSE62452, GSE57495, and GSE17891 datasets revealed that low expression of ITGα3 significantly improved median survival (MS) compared with a high expression of ITGα3 in patients with pancreatic cancer (Fig. 4A–F). To further analyze the detailed role of ITGα3 in prognosis of patients with pancreatic cancer, we subdivided PACA-AU, PAAD-US-TCGA, and GSE79668 datasets into different age groups. The highly ITGα3-expressing group at all ages showed significantly worse median survival than the poorly expressing group. Group of over 50 years had the worst median survival (MS: 393 days) in GSE79668 dataset, followed by those of over 60 years (MS: 481 days) in PAAD-US-TCGA dataset and those who were below 70 years (MS: 537 days) in PACA-AU dataset (Table 2).

**Table 2:** Clinical outcomes correlated with integrin α3 (ITGα3) expression levels.

data-set-ID	stratification	*P* value	Low (MS)	High (MS)	Low (N)	High (N)
PACA-AU	ALL	0.00163884	1874	537	39	40
PACA-AU	AGE>30	0.00258695	1874	709	39	39
PACA-AU	AGE>40	0.00248286	1874	709	38	39
PACA-AU	AGE>50	0.0082042	1874	709	35	38
PACA-AU	AGE>60	0.0238491	1874	709	29	30
PACA-AU	AGE<70	0.00205248	1144	537	23	23
PACA-AU	AGE<80	0.031809	1874	1021	35	38
PAAD-US-TCGA	ALL	0.0336586	666	598	69	69
PAAD-US-TCGA	AGE>30	0.0336586	666	598	69	69
PAAD-US-TCGA	AGE>40	0.0323066	652	511	68	69
PAAD-US-TCGA	AGE>50	0.0113558	603	511	62	62
PAAD-US-TCGA	AGE>60	0.00818206	1332	481	48	49
PAAD-US-TCGA	AGE<70	0.0450421	1332	511	43	45
PAAD-US-TCGA	AGE<80	0.0078225	1332	598	63	63
GSE79668	ALL	0.000233238	702	382	25	26
GSE79668	AGE>30	0.0011241	647	371	25	25
GSE79668	AGE>40	0.0011241	647	371	25	25
GSE79668	AGE>50	0.00358437	601	341	22	22
GSE79668	AGE>60	0.0115516	611.5	342	17	18
GSE79668	AGE<70	0.00645222	702	449	17	17
GSE79668	AGE<80	0.000351786	702	393	25	25

^Ɨ^Low (MS) indicates the median survival of low expressed ITGα3 group.

^Ɨ^High (MS) indicates the median survival of high expressed ITGα3 group.

^Ɨ^Low (N) indicates the number of patients with low expression of ITGα3.

^Ɨ^High (N) indicates the number of patients with high expression of ITGα3.

The English in this document has been checked by at least two professional editors, both native speakers of English.

These results strongly indicate that low expressed ITGα3 improves the malignancy of human pancreatic cancer.

## Discussion

Thorough knowledge underlying malignant pancreatic cancer features has become increasingly critical due to poor clinical outcome of patients with this disease. Evidently, survival rates of pancreatic cancer patients have not improved during the past few decades despite continuous efforts to improve their prognosis^1^. This grave convalescence might originate from the strong resistance of this cancer to chemotherapies, instinct metastasis to different nests, and hard to obtain early diagnosis^17–19^.

Results of the present study demonstrate that ITGα3 has critical functions in human pancreatic cancer features *via* adducing *in silico*-*in vitro*-*in vivo*-clinical evidences. To the best of our knowledge, this is the critical study shows that blockade of ITGα3 might be a promising strategy to inhibit malignant pancreatic cancer through ablating the strict connective signalling pathway involving epidermal growth factor receptor (EGFR).

Integrins (ITGs) play a critical role in metastasis to evade apoptosis and maintain cellular motility. It has been reported that ITGs help tumour cells acquire malignant features via interactions with their corresponding ECM components^20–22^. Integrin α3 (ITGα3) is a receptor for ECM molecules such as fibronectin, laminin- 5, -10, or -11^23^. Being a pivotal player in aggressive cancer phenotypes, α3β1 integrin plays an oncogenic role in different cancer types. It is critically involved in membrane protrusion of U251MG glioblastoma cells^24^ and epithelial mesenchymal transition (EMT) in breast cancer cells^25^. Consistent with previous reports, the current study also revealed the unique function of ITGα3 in human pancreatic cancers. All data were retrieved from the public GEO database indicated that *ITGα3* was specifically over-expressed in pancreatic cancers compared with normal pancreas. The expression of ITGα3 was also confirmed in human pancreatic cancer tissues and cell lines. In contrast, it had very weak expression level in normal pancreas cells based on Western blot analysis. Of note, ITGα3 was shown to be associated with aggressive phenotypes of human pancreatic cancers. Ablation of ITGα3 expression using si-RNA transfection significantly inhibited viability and migration of human pancreatic cancer cells, indicating that silencing ITGα3 expression triggered prominent anti-cancer effects independent of its interactions with various ECM components. A recent clinical trial of gene therapy suggested remarkable therapeutic benefits and an excellent safety record^26^, prompting serious consideration of blockade of *ITGα3* via innovative gene therapy approaches. In addition, effective small-molecule inhibitors or neutralizing antibody against ITGα3 should be developed as pancreatic cancer therapy. Based on CCLE database, there were mostly negative correlations (75%, 16/24 cases) between *ITGα3* expression and various anti-cancer drugs. Interestingly, clinically approved drugs for human pancreatic cancer such as paclitaxel, irinotecan, and erlotinib^27^ all showed negative correlations with *ITGα3* expression, indicating that ITGα3 might be potentially involved in chemo-resistance of pancreatic cancer. However, substantial correlations of *ITGα3* expression and various anti-cancer drugs should be verified.

Altered levels of EGFR play an important role in the growth, invasion, adhesion, and angiogenesis of numerous cancers. It has been reported that the EGFR signalling induces tumour growth, invasion, adhesion, and angiogenesis^12^. Consistent with previous reports, the results of the present study suggest that EGFR signalling was regulated by ITGα3 expression *in silico* and *in vitro* for the first time. It is noteworthy that the protein level of EGFR, but not the mRNA level, is regulated by silencing ITGα3 expression, indicating that ITGα3 affects EGFR post-transcriptional processes.

Natural regulator of negative-feedback regulation in *de novo* EGFR expression, LRIG1 is a cell-surface transmembrane protein, which contains a leucine-rich repeat (LRR) domain and interacts with EGFR under ligand-independent conditions^28,29^. The natural structure of LRIG1 is sufficient for direct binding to the extracellular region of EGFR. Therefore, the intracellular domain of EGFR is dispensable for the formation of EGFR-LRIG1 complex and the LRIG1 is capable of directing ubiquitylation and degradation of EGFR^28^. Notably, ITGα3 silencing led to a reduction in EGFR expression levels *via* induction of functional LRIG1 expression. Based on our *in-silico* results correlating *IFIs* and *ITGα3*, the LRIG1 expression was enhanced by blockade of ITGα3 expression, implying that ITGα3 might act as “the sealing” for protection of EGFR against natural inhibitor, LRIG1. However, understanding these relationships will positively stimulate our cogitation to design an effective therapy for pancreatic cancer.

Notably, the *in vivo* anti-cancer effects of ITGα3 targeting were observed in the presence of an adequate number of each group (control: n = 6, sh-ITGα3: n = 12). Blockade of ITGα3 expression evoked anti-cancer effect by inhibiting EGFR signalling pathways. Selective down-regulation of EGFR expression was also demonstrated in sh-ITGα3 xenograft models compared with sh-control xenograft models. Therefore, the results of the present study showed that EGFR signalling can be controlled by ITGα3 expression through *in vivo* xenograft models.

A previous study reported the involvement of ITGα3 in clinical outcomes of colon and pancreatic cancers^9,30^. However, the clinical significance of ITGα3 in prognosis remains controversial. Miyamoto *et al*. suggested that poorly expressed ITGα3 is related to unfavourable prognosis^31^. In contrast, another study suggested that the diagnostic levels of integrin α3, β4, and β5 gene expression determine the prognosis of tongue squamous cell carcinoma^22^. Notably, our results strongly suggested that ITGα3 expression had clinical significance in pancreatic cancer. Low expression of ITGα3 significantly improved the prognosis of pancreatic cancer patients compared with high expression of ITGα3 according to independent PACA-AU, PAAD-US-TCGA, GSE79688, GSE62452, GSE57495, and GSE17891 datasets. Moreover, PACA-AU, PAAD-US-TCGA, and GSE79688 datasets were analyzed in further detail in different age groups. All the datasets showed that high ITGα3 expression significantly aggravated the prognosis of pancreatic cancer patients compared with low expression of ITGα3 according to age, although the significance was not considerable in older ages. To our knowledge, this is a reliable finding showing that ITGα3 expression has clinical significance in human pancreatic cancer.

Collectively, the results of the present study indicate that ITGα3 plays a pivotal role in human pancreatic malignancy based on *in silico*, *in vitro*, *and in vivo*-clinical evidence. Targeting ITGα3 might be a promising strategy to inhibit malignant pancreatic cancer by ablating the EGFR signalling pathway.

## Material and Methods

### Gene expression profile

Microarray analysis was performed from Gene Expression Omnibus (GEO) public microarray database at NCBI (https://www.ncbi.nlm.nih.gov/geo/). Histological type of samples were designated as normal pancreas or pancreatic tumour according to its annotation in GEO as described previously^32^. Correlations between *ITGα3* mRNA expression and expression levels of *EGFR* were analysed in GEO public microarray database using GraphPad Prism version 5.01 for Windows (San Diego, CA, USA). Correlations between *ITGα3* expression and various anti-cancer drugs were demonstrated using Cancer Cell Line Encyclopedia (CCLE, https://www.broadinstitute.org/ccle/) public database as described previously^33^.

### Cell culture and reagents

Human pancreatic cancer cells, AsPC-1 (#21682), Capan-1 (#30079), Capan-2 (#30080), Miapaca-2 (#21420), Panc-1 (#21469), SNU-213 (#00213), and SNU-410 (#00410) were purchased at Korean Cell Line Bank (KCLB, Seoul, Korea). CFPAC-1 (CRL-1918) was purchased from American Type Culture Collection (ATCC, Manassas, VA, USA). These cells were maintained as described previously^32^. H6c7 (ECA001) was obtained from Kerafast (Boston, MA, USA) and grown as described previously^34^. Monoclonal antibody for ITGα3 (sc-374242) was purchased at Santa Cruz Biotechnology (Santa Cruz, CA, USA). Antibodies against epidermal growth factor receptor (EGFR, #4267), phospho-EGFR (Tyr1068, #3777), mitogen-activated protein kinase kinase 1/2 (MEK1/2, #4694), phospho-MEK1/2 (Ser217/221, #9154), protein kinase B (AKT, #9272), phospho-AKT (Ser473, #9271), extracellular signal-regulated kinase1/2 (ERK1/2, #9107), phospho-ERK1/2 (Thr202/204, #9106), leucine-rich repeats and immunoglobulin-like domain protein 1 (LRIG1, #12752), receptor-associated late transducer (RALT, #2440), caspase-3 (#9665), cleaved caspase-3 (#9664), and glyceraldehyde-3-phosphate dehydrogenase (GAPDH, #5174) were obtained from Cell Signaling Technology (Beverly, MA, USA). Recombinant EGF (#236-EG) was purchased at R&D Systems (Minneapolis, MN, USA).

### Transfection with small interfering RNA (si-RNA)

Transfection of siRNAs was performed using Effectene reagent (Qiagen, Hilden, Germany) as described previously^35^. Oligonucleotides specific for ITGα3 (sc-35684 for #1 and SDH-1002 for #2) and scrambled control (sc-37007) were obtained from Santa Cruz Biotechnology and Bioneer (Daejeon, Korea), respectively. The effect of si-RNA transfection was validated by Western blotting of ITGα3 protein.

### Phospho-RTK array

To demonstrate intracellular signalling by si-ITGα3 transfection in AsPC-1 cells, a phospho-RTK array kit (ARY001B, R&D Systems, Minneapolis, MN, USA) was used according to the manufacturer’s instructions.

### Measurement of cell viability

To evaluate cell viability after si-ITGα3 transfection, WST-1 (#11644807001, Sigma-Aldrich, St. Louis, MO, USA) assay was performed as described previously^36^.

### Trans-well migration assay

Migration assay was demonstrated using a Trans-well apparatus (#3422, Corning, Corning, NY, USA) as described previously^33^. Briefly, cells were transfected with scrambled si-RNA or si-ITGα3 for 72 h under normal cultured condition. Then, cells were applied to the upper-chamber containing RPMI without serum for 6 h, and cells that had migrated to the back side of the filter were stained. The eluted dye using 10% acetic acid was measured at 560 nm in an enzyme-linked immunosorbent assay (ELISA) reader (Bio-Rad, Richmond, CA, USA).

### Western blot analysis

To determine protein levels of ITGα3 in eight pancreatic cancers and H6c7 cells, Western blot analysis was demonstrated as described previously^37^. Bands were subjected to densitometry analysis using ImageJ software (National Institutes of Health, Bethesda, MD, USA).

### RNA preparation and quantitative real-time polymerase chain reaction

Total RNA extraction was performed using TRIzol reagent (Invitrogen, Carlsbad, CA, USA) and diverted to cDNA using a RNA PCR kit (Takara Bio Inc, Japan) as described previously^38^. Quantitative real-time PCR analysis was demonstrated using a StepOne Real-Time PCR system (Applied Biosystems) according to the manufacturers’ protocols. Primers sequences for quantitative real-time PCR for human ITGα3 (P205125V) and EGFR (P187403V) were obtained from Bioneer (Daejeon, Korea) Expression normalization was performed using GAPDH level as described previously^39^. Primer sequences for quantitative real-time PCR were as follows: GAPDH (forward primer, 5′-TCACTGGCATGGCCTTCCGTG-3′; reverse primer, 5′-GCCATGAGGTCCACCACCCTG-3′).

### Generation of the ITGα3-specific short hairpin (sh)RNA stable AsPC-1 cell line

Plasmids specific for sh-ITGα3 and control shRNA (sc-35684-SH and sc-108060) were purchased at Santa Cruz Biotechnology to stably suppress ITGα3 expression using a sh-activated gene silencing vector system. Briefly, one day after transfection with shRNA constructs, AsPC-1 cells were grown in Dulbecco’s complete medium containing 5 µM puromycin for 3 days to select the stable transfectants.

### Xenograft tumour model

Nude mice (BALB/c) were purchased at Orient (Seongnam, Korea) at 6–8 weeks of age. sh-control/AsPC-1 (1 × 10^7^) and sh-ITGα3/AsPC-1 cells (1 × 10^7^) were subcutaneously injected into the right flank as described previously^40^. Body weight was recorded about every 3 days. *In vivo* experiments were carried out in accordance with guidelines approved by the Animal Bioethics Committee (ABC) of Jeju National University (approval number: 2016-0049).

### GSE data-set analysis

mRNA expression profiles was obtained from the Gene Expression Omnibus (GEO) public microarray data-base. We independently integrated data sets obtained from several groups using the absolute normalization method SCAN.UPC^41^. Then, we restricted the integration to Affymetrix Human Genome U133 Plus 2.0 Array platform (GPL570) because the normalization method is dependent on the total number of probes. All data were normalized by the default option of SCAN.UPC. Eight data sets were used: GSE9599, -15471, -16515, -17891, -32676, -39409, -42952, and -46385. Each probe was converted to EntrezID. Several probes for the same EntrezID were averaged. Quantile-quantile normalization was applied to all samples to remove batch effects. To test prognostic value of a gene, samples were divided into two groups using median gene expression level as threshold. Log-rank test was then performed using Graph Prism version 5.

### Statistical analyses

All data are presented as mean ± standard deviation. Student’s t-test was used to determine significance level for comparison between two independent samples. Groups were compared by one-way analysis of variance (ANOVA) with Tukey’s *post hoc* test for significant main effects using SPSS 12.0 K for Windows (SPSS Inc., Chicago, IL, USA).

## Supplementary information

Dataset1

## Data Availability

Additional data are available as Supplementary information.
