# Supplementary material for: Blockade of integrin α3 attenuates human pancreatic cancer via inhibition of EGFR signalling
Source: Sci Rep. 2019 Feb 26;9:2793. doi: 10.1038/s41598-019-39628-x (PMC6391393; doi:10.1038/s41598-019-39628-x)
Supplement: Supplementary file 1 — Dataset1 [file 41598_2019_39628_MOESM1_ESM.docx]

**Blockade of integrin α3 attenuates human pancreatic cancer via inhibition of EGFR signalling**

**Authors and Affiliations**

Jungwhoi Lee ^1^*, Jungsul Lee^3^, Chulhee Choi^3^, and Jae Hoon Kim ^1, 2^

^1^ Department of Applied Life Science, SARI, Jeju National University, Jeju-do 690-756, Republic of Korea

^2^ Subtropical/tropical Organism Gene Bank, Jeju National University, Jeju-do 690-756, Republic of Korea

^3^ Department of Bio and Brain Engineering, KAIST, Daejeon 34141, Republic of Korea

^4^ Cellex Life Sciences Inc., Daejeon 34141, Republic of Korea

**Requests for reprints**

*Address correspondence and reprint requests Jungwhoi Lee, Department of Biotechnology, College of Applied Life Science, Jeju National University, 102 Jejudaehak-ro, Jeju-si, Jeju-do 690-756, Republic of Korea. Tel: +82-64-729-8556; Fax: +82-64-756-3351; E-mail: sdjd1108@kaist.ac.kr

**Supplementary Fig. 1.** AsPC-1, Miapaca-2, and Panc-1 cells were transfected with scrambled or ITGα3-specific siRNA. After 72 h of transfection, the cell lysates were subjected to Western blot using antibodies specific for ITGα3 and GAPDH.

**Supplementary Fig. 2.** **Functional** **integrin α3 (ITGα3) expression in pancreatic cancer**

(A) AsPC-1, Miapaca-2, and Panc-1 cells were transfected with scrambled or ITGα3-specific siRNA (#2) for 72 h under serum-free cultured conditions. The viability was measured by WST-1 assay (*n* = 3; Tukey’s *post-hoc* test was used to detect significant differences in ANOVA, p < 0.0001; asterisks indicate a significant difference compared with 0% inhibition, ***P* < 0.01). (B) AsPC-1, Miapaca-2, and Panc-1 cells were transfected with scrambled or ITGα3-specific siRNA (#2). After 48 h of transfection, the cells were exposed to serum-starved conditions. After 24 h of serum starvation, migrated cells were evaluated using the Transwell-migration assay (*n* = 3; Tukey’s *post-hoc* test was used to detect significant differences in ANOVA, p < 0.0001; asterisks indicate significant differences compared with 0% inhibition, ***P* < 0.01, ****P* < 0.001).

**Supplementary Fig. 3.** Correlation of *ITGα3* and *EGFR* expression in human pancreatic cancer cells

AsPC-1 cells were transfected with scrambled or ITGα3-specific siRNA for 48 h. *ITGα3* and *EGFR* mRNA expression levels were measured by quantitative real-time PCR (a.u. indicates arbitrary units using values of normalization to *GAPDH.* Data is representative of three individual experiments).

**Supplementary Fig. 4**. The correlation between *ITGα3* and *inducible feedback inhibitors* (IFIs) expressions in pancreatic cancer

A-B, The correlations between *ITGα3* and *SOCS4* (A) or *SOCS5* (B) expression in pancreatic cancer samples were calculated using the Gene Expression Omnibus (GEO) public microarray database (Pearson’s correlation coefficient (PCC) was used for statistical analysis).

**Supplementary Fig. 5**. EGFR proteins in various human pancreatic cancer cells and H6c7 cells were detected by Western blot. GAPDH was measured as a control. Data is representative of three individual experiments.

**Supplementary Fig. 6**. ***In vitro* characterization of the short hairpin-Integrin α3 (**ITGα3**) AsPC-1 cell line**

(A) ITGα3 expression levels were evaluated by Western blot analysis using sh-control and sh-ITGα3 AsPC-1 cells. GAPDH was used for loading control. Data is representative of three individual experiments. (B) Cell viability was measured by the WST-1 assay in sh-control and sh-ITGα3 AsPC-1 cells under a serum-starved condition (*P*-value by Student’s *t* test, ***p* < 0.01). Migrated cells were evaluated using the Transwell-migration assay using sh-control and sh-ITGα3 AsPC-1 cells (*P*-value by Student’s *t* test, ***p* < 0.01).

**H6c7**
